# Selective Daily Mobility Bias in the Community Food Environment: Case Study of Greater Hartford, Connecticut

**DOI:** 10.3390/nu15020404

**Published:** 2023-01-13

**Authors:** Ailing Jin, Xiang Chen, Xiao Huang, Zhenlong Li, Caitlin E. Caspi, Ran Xu

**Affiliations:** 1 Department of Geography, University of Connecticut, Mansfield, CT 06269, USA; 2 Department of Allied Health Sciences, University of Connecticut, Mansfield, CT 06269, USA; 3 Department of Geosciences, University of Arkansas, Fayetteville, AR 72701, USA; 4 Department of Geography, University of South Carolina, Columbia, SC 29208, USA; 5 Rudd Center for Food Policy and Health, University of Connecticut, Hartford, CT 06103, USA

**Keywords:** obesity, community food environment, human mobility, selective daily mobility bias (SDMB), socio-economic characteristics

## Abstract

The community food environment has potential influences on community members’ dietary health outcomes, such as obesity and Type II diabetes. However, most existing studies evaluating such health effects neglect human mobility. In food patrons’ daily travels, certain locations may be preferred and patronized more frequently than others. This behavioral uncertainty, known as the selective daily mobility bias (SDMB), is less explored in community-food-environment research. In this paper, we aim to confirm the existence of the SDMB by systematically exploring the large-scale GPS-based restaurant-visit patterns in the Greater Harford region, Connecticut. Next, we explore the restaurant and neighborhood characteristics that are associated with the restaurant-visit patterns. Our primary results demonstrate that (1) most restaurant customers originate from areas outside of the census tract where the restaurant is located, and (2) restaurants located in socially vulnerable areas attract more customers in total, more customers from local areas, and more customers from other socially vulnerable areas. These results confirm the relevance of the SDMB to the community food environment, and suggest ways that the SDMB can be moderated by an uneven socio-economic landscape. The findings demonstrate the necessity of incorporating human-mobility data into the study of the community food environment.

## 1. Introduction

Obesity is considered a widespread chronic disease, currently affecting 41.9% of adults over the age of 20 and 19.7% of children and adolescents in the United States (US) [1]. Factors in the built environment, such as access to less healthy food-retail and restaurants, are considered environmental drivers behind the development of obesity [2,3,4,5,6,7,8,9]. However, findings on the relationship between community food environments and obesity are equivocal. For example, some studies found a positive relationship between fast-food restaurant access and obesity, implying that easy access to fast-food restaurants would likely induce obesity cases [3,4,5,6], while others found negative [7], null [8], and mixed relationships [9]. A review article found that while many studies identified positive correlations between fast-food restaurants and obesity indicators in the same neighborhood, this correlation was not identified in nearly half of the studies under review [2,10].

While such inconsistencies could be induced by the heterogeneity of the measurement, such as measures of environmental exposures, units of analysis, and obesity indicators, one methodological limitation in previous studies is the neglect of human mobility [11]. Specifically, most studies have employed predefined administrative units (e.g., counties, census tracts) for analysis, assuming that people are only exposed to food retailers within an arbitrary administrative unit based on, for example, where they live [2]. However, people’s daily activities are not limited to a single analysis unit or the neighborhood they live in, but can expand to external regions [12]. In addition, people make active choices on a daily basis which reflect the locations they prefer and patronize [13]. Thus, it is uncertain if diet-related health outcomes (e.g., obesity) are primarily driven by food-environmental exposure or people’s active choices to go to certain places. This issue, known as the selective daily mobility bias (SDMB) [13,14], can lead to the unequal obesogenic roles that food retailers play while obfuscating obesogenic drivers in the food systems.

The SDMB originates from the concept of the “selective mobility bias (SMB)”, which focuses on selective residential migration, meaning that people intentionally select themselves into different neighborhoods based on various household and neighborhood characteristics [15,16]. The SDMB expands this concept to reflect the selective bias in peoples’ daily visited sites [17]. While the SDMB is an emerging issue that has been observed and identified across multiple contexts (e.g., choice of transportation mode, travel route, travel distance, daily travel time, facilities, and general choice of destinations in daily life [18,19,20,21,22,23,24,25,26]), to date, the SDMB has received scant attention in community food environmental research [15]. In addition, the issue has not been well discussed in the context of health inequity, meaning that people with different socioeconomic statuses could be affected by the SDMB unequally in their everyday procurement of food.

To account for the SDMB, researchers have relied on surveys and interviews to study residents’ choices and visits to food retailers [27]. However, these tools are usually time-consuming, not easily scalable, and can suffer from recall bias [28]. With the wide adoption of location-based services (LBS) such as GPS-tracking, researchers are able to study the SDMB in a scalable and cost-effective manner in terms of tracking peoples’ daily activity locations and trajectories [29]. Several studies have employed these LBS technologies to study the SDMB in a food environment context [30,31,32,33]. However, most GPS-based studies suffer from small sampling sizes and short timeframes, which limits their potential applications on a large scale.

To overcome the limitations of previous studies using GPS tracking and demonstrate the SDMB in the community food environment, this study adopts a large-scale human-mobility dataset collected from GPS-enabled smartphones in the US. We evaluate the visit pattern of Food-Away-From-Home (FAFH) in the Greater Hartford region, Connecticut. Specifically, we focus on the visit of full-service and fast-food restaurants, which are considered the main sources of FAFH, and then relate the visit patterns to the risk factors for poor diet and obesity development [34]. We then analyze various factors (i.e., the characteristics of the restaurant and the census tract where the restaurant is located) associated with the restaurant-visit patterns, with a specific focus on the socio-economic landscape of the neighborhood. To this end, this case study not only exhibits the SDMB in the community food environment but also demonstrates the potential to incorporate large-scale human-mobility data to assist health policymaking and health promotion.

## 2. Materials and Methods

### 2.1. Sample and Study Design

We conducted an observational study in the Greater Hartford region, Connecticut, US. Hartford is the fourth most populous city in Connecticut, with a significant minority population [35]. As Hartford has intensive spatial interaction with surrounding areas in terms of traffic, human movements, and services, we expanded our study area to Hartford and its five adjacent towns, including Bloomfield, West Hartford, East Hartford, Newington, and Wethersfield.

**Figure 1:**
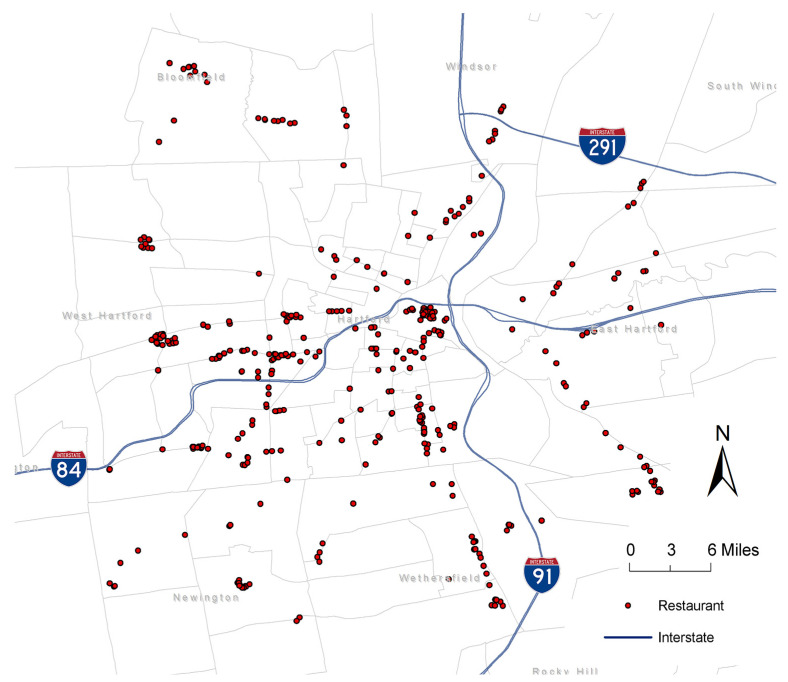
Spatial distribution of restaurants in the Greater Hartford region.

The mobility data were sourced from SafeGraph, a data company that aggregates anonymized location data from mobile devices to provide insights into physical places [36,37]. We used the Safegraph’s Core Places and Patterns datasets, which aggregated data from approximately 10% of all the mobile devices in the US and contained information about the number of visits from people’s home census tract to each point of interest (POI). The data were made publicly available during the early stage of the Coronavirus Disease 2019 (COVID-19) pandemic. SafeGraph uses a validated algorithm to determine visits to POI, and the duration of the visit must last for at least 4 minutes to count as a visit to a given POI [37,38]. SafeGraph defines a person’s “home” to be the location where the mobile device is detected the most at night (i.e., from 18:00 to 07:00) over a 6-week period, and each location is defined at the Geohash-7 level (153 × 153-m grid). According to SafeGraph, no privacy rights were violated during data collection; the data do not contain individual information; and the data cannot be “de-anonymized” using any known method of re-identification [39]. Previous studies showed that the mobile-device sample in SafeGraph is generally representative of the general population in terms of sociodemographic variables (e.g., racial/ethnic composition, educational attainment, and income) and the overall US Census population count [40,41]. Using SafeGraph data, we included all restaurant POIs in the Greater Hartford region. After validating the restaurant locations and removing duplicates, we included a total of 396 restaurants, where 69 were full-service restaurants (defined by the North American Industry Classification System [NAICS] code 722211) and 327 were limited-service restaurants (NAICS code 722513). A cross-validation with restaurant directories from Yelp and Google Place showed that the SafeGraph data included more than 80% of the restaurant POIs in the study area. The 396 restaurants included in this study were visualized in ESRI ArcGIS Pro, as shown in Figure 1. 

The raw visit data from SafeGraph contained 250,112 records, and each record indicated the number of visits from a home census tract to a POI in a given month in 2018–2019. If there were 1–4 visits from a home census tract to a POI in a given month, the number of visits was coded as 4 to protect privacy. As most of the visits were concentrated within Connecticut and a significant portion of the visits from home census tracts outside Connecticut were coded as 4 in the SafeGraph data, we focused on all home census tracts within Connecticut. We further aggregated all visit data over time, and the final data for analysis included 66,605 visit records, with each record containing the destination (restaurant POI), origin (people’s home census tract), and the total number of visits in 2018–2019. It is worth mentioning that since the study period was before the COVID-19 outbreak, the data can represent the general mobility patterns.

### 2.2. Measures

*Visit Pattern.* We aggregated the SafeGraph mobility data at the restaurant level. For each restaurant, we calculated (a) the median distance that the restaurant customers traveled, (b) the percentage of customers originating from the same census tract where the restaurant was located, (c) the percentage of customers originating within a 1-mile radius of the restaurant location, and (d) the total number of visits in 2018–2019.

*Restaurant characteristics.* We categorized the restaurants into full-service restaurants and limited-service restaurants, based on their NAICS code. In addition, we retrieved various restaurant characteristics from Yelp (up to 2019), including the number of reviews, ratings (1–5), and the average cost per person for a meal (1–3) (see details in Table 1).

*Neighborhood characteristics.* We obtained the characteristics of the customers’ home census tracts and the census tracts where restaurants are located from multiple sources. For each census tract, we obtained the social vulnerability index (SVI) in 2018 from CDC [42]. The SVI ranks each census tract, based on 15 sociodemographic factors which are originally derived from the 2014–2018 American Community Survey 5-Year Estimates. These factors can be categorized into four themes—socioeconomic status, household composition and disability, minority status, and housing-type and transportation. We obtained the overall percentile ranking of SVI (ranging from 0 to 1, where 1 means the most socially vulnerable) of the census tract where each restaurant is located. For each restaurant, we also calculated the overall SVI percentile-ranking of the customers’ home census tracts (weighted by the number of visits).

In addition, we collected the population counts for each census tract from the 2020 decennial censuses [43], and the food-desert label and urban-tract label from the United States Department of Agriculture (USDA) [44].

### 2.3. Analysis

To illustrate restaurant-visit patterns, we first performed visit-flow visualizations in ArcGIS, and then we summarized the percentage of customers originating from the census tract where each restaurant was located, as well as the percentage of customers originating from a radius of 1, 1 to 5, 5 to 10, and 10 to 20 miles of the restaurant location, separately. To explore how visit patterns differed by socioeconomic characteristics of the restaurant area, we further examined the restaurant-visit patterns for restaurants in the top 10% socially vulnerable areas (measured by the SVI) in our data.

To explore how neighborhood and restaurant-level characteristics were associated with the restaurant-visit patterns, we performed separate multivariate linear regressions at the restaurant level, with five outcomes: (a) the percentage of customers who originated from the same census tract where the restaurant was located, (b) the percentage of customers who originated within a 1-mile radius of the restaurant location, (c) the overall SVI percentile-ranking of the visitors’ home census tracts (weighted by the number of visits), (d) the total visit-count of the restaurant (log-transformed), and (e) the median distance customers traveled to get to each restaurant. In each regression, independent variables included restaurant characteristics (i.e., rating, price, log-transformed review counts, and restaurant category), and characteristics of the census tract where the restaurant was located (i.e., total population, overall SVI percentile-ranking, food-desert label, and urban-tract label).

## 3. Results

### 3.1. Restaurant-Visit Patterns

**Figure 2:**
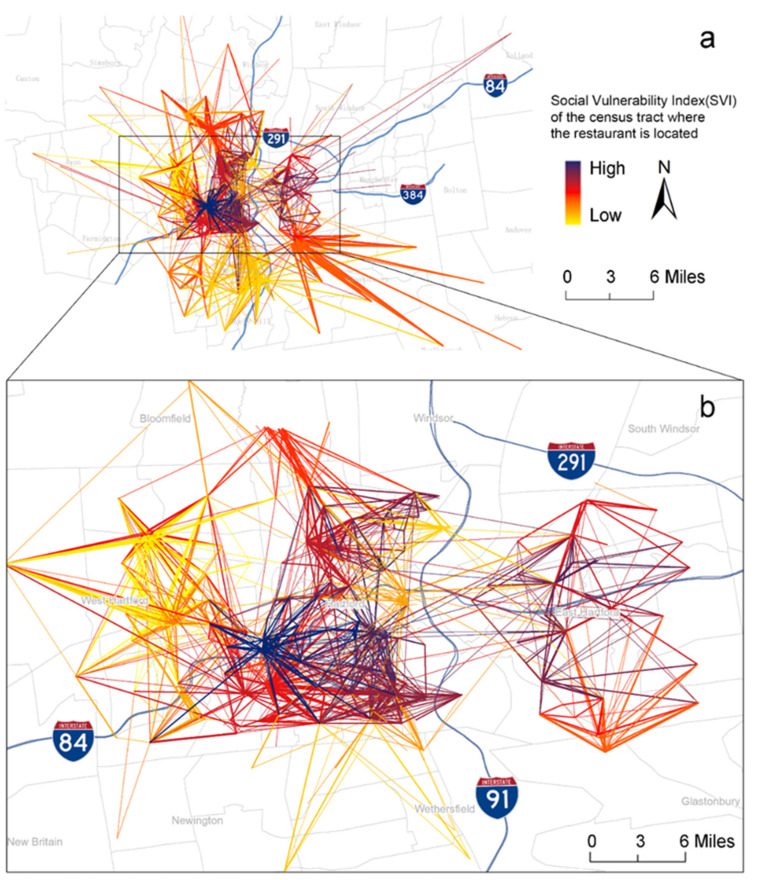
Restaurant-visit patterns in the Greater Hartford region. (**a**) shows overall restaurant-visit patterns in the Greater Hartford region, and (**b**) zooms into East Hartford, West Hartford, and Hartford. Each line connects a restaurant to a census tract. Line thickness indicates the number of visits; line color indicates the percentile-ranking of SVI for the census tract where the restaurant is located (darker means more socially vulnerable). Only relations with more than 41 visits (top 25% percentile) are included in the figure.

Figure 2 depicts the overall restaurant-visit patterns in the study area. The figure shows that a significant portion of the restaurant visits were from areas beyond the census tract where the restaurant was located. Spatial descriptive analyses further showed that on average only 8.4% (standard deviation [SD] = 5.8%) of the customers originated from the restaurant’s census tract; on average, 18.9% (SD = 11.8%), 43.5% (SD = 10.3%), 17.4% (SD = 5.8%), and 13.2% (SD = 6.6%) of the customers originated from within a radius of 1, 1 to 5, 5 to 10, and 10 to 20 miles, respectively, of the restaurant location; on average, customers traveled a median distance of 2.9 (interquartile range [IQR = 2.3–4.4] miles to patronize the restaurant.

Furthermore, Figure 2 shows that there was considerably less mobility in terms of restaurant visits in more socially vulnerable areas (i.e., higher SVI, dark-colored lines), and that restaurants in these high SVI areas mostly attracted visitors from nearby. However, a further spatial descriptive analysis showed that even in the top 10% most socially vulnerable areas, there were still considerable journeys originating from external tracts: only 9.7% (SD = 4.9%) of the customers originated from the restaurant’s census tract; on average, 28.7% (SD = 9.0%), 39.8% (SD = 6.1%), 14.9% (SD = 3.5%), and 10.3% (SD = 4.4%) of customers originated from within a radius of 1, 1 to 5, 5 to 10, and 10 to 20 miles, respectively, of the restaurant; on average customers, still traveled a median distance of 2.0 [IQR = 1.6–2.8] miles to patronize the restaurant.

### 3.2. Factors Associated with the Restaurant-Visit Patterns

Descriptive statistics of all the variables used in the restaurant-level analysis are summarized in Table 1. The table shows restaurants included in the study were mostly limited-service restaurants (82.58%), located in urban areas (99.24%), not in the food-desert census tract (71.21%), and within a cost range of between USD 11 and 30 (52.53%) per person for a meal. The average review-counts and the average rating were 86.00 (SD = 132.00) and 3.50 (SD = 0.85), respectively. The distribution of the overall SVI percentile-ranking of the customers’ home census tracts (mean (SD) = 0.61 (0.14)) was similar to the overall SVI percentile-ranking of the census tract where the restaurant was located (mean (SD) = 0.59 (0.29)). The median total number of visits recorded in SafeGraph was 2204 (IQR [1292–3593]) for a restaurant in 2018–2019.

Multivariate-linear-regression results are summarized in Table 2. Neighborhood characteristics of the restaurant location were associated with visit patterns. Specifically, the SVI of the census tract where the restaurant was located had strong associations with many visit patterns. A one-percentile increase in overall SVI ranking (more socially vulnerable) in the restaurant’s census tract was associated with a decrease of 0.0196 (95% confidence interval [CI]: 0.0125, 0.0267) miles in the median distance customers traveled, an increase of 0.1661% (95% CI: 0.1251, 0.2072) of customers originating within a 1-mile radius of the restaurant location, an increase of 0.45% (95%CI: 0.05, 0.84) in the total visit-count, and an increase of 0.28 (95% CI: 0.25, 0.32) in the overall SVI percentile-ranking of the customers’ home census tracts (results were robust after controlling for distance traveled). In addition, restaurants in food-desert tracts drew 2.48% (95% CI: 1.13, 3.83) more customers from the same census tract, and the overall SVI ranking of the customers’ home census tracts was 4 percentiles (95% CI: 1, 6) higher for those restaurants.

In terms of restaurant characteristics, we did not observe significant differences in visit patterns by restaurant category or price. The number of restaurant reviews on Yelp (log-transformed) had a negative association with the percentage of customers from the same census tract as the restaurant (β = −0.92 (95% CI: −1.35, −0.50)), the percentage of customers originating within a 1-mile radius of the restaurant location (β = −2.33 (95% CI: −3.16, −1.50)), and the overall SVI percentile-ranking of the customers’ home census tracts (β = −0.02 (95% CI: −0.03, −0.01)). Additionally, the number of restaurant reviews had a positive association with the median distance traveled (β = 0.33 (95% CI: 0.18, 0.47)) and the total number of visits (β = 0.17 (95% CI: 0.09, 0.25)). Lastly, restaurant rating was positively associated with the percentage of customers coming from the same census tract as the restaurant (β = 0.81 (95% CI: 0.18, 1.44)) but was negatively associated with the total number of visits (β = −0.25 (95% CI: −0.37, −0.14)).

## 4. Discussion

This study demonstrated the existence of the SDMB in the community food environment in relation to restaurant visits. Our study has two primary findings. First, the significant out-of-home tract visit-patterns reveal that the majority of the restaurant’s customers did not originate from the same census tract where the restaurant was located. Second, we found important restaurant and neighborhood characteristics that were associated with restaurant-visit patterns. Specifically, the SVI of the census tract where the restaurant was located played an important role—restaurants located in socially vulnerable areas not only attracted more customers in general, they also attracted more customers from local areas, as well as customers from other socially vulnerable areas.

By using large-scale human-mobility data, this study is one of the first to systematically demonstrate the existence of the SDMB in restaurant visits on the community level. Our results are in line with previous findings demonstrating the wide existence of the SDMB across various domains, such as choice of transportation mode, travel route, travel distance, daily travel-time, sports facility and general destinations in daily life [18,19,20,21,22,23,24,25,26]. For example, several studies examined the influence of individuals/households sociodemographic factors on travel route and mode [22], daily travel-time [25], and travel distance [26], and found peoples’ travel behaviors differ according to individual characteristics. In addition, previous studies observed that individuals make intentional choices of sport facilities for physical activity, which often go beyond their residential neighborhood [19,20]. Another study found individual environmental preferences, such as green environments, can result in differences in travel route and mode selection [22]. While the manifestation of the SDMB in food environment research is emerging, most of the existing studies do not empirically evaluate the SDMB or consider it as a potential limitation [15]. They suggest that ignoring the SDMB could lead to biased estimation and erroneous conclusions about the impact of food-environment exposure on food choices and dietary-related health outcomes [15]. Such bias can lead to the overestimation of the association between spatial accessibility and the actual use [15,45]. Our first finding is in line with previous research, showing that people’s activity space and access to food extended beyond residential neighborhoods, and there were significant differences between the place-based residential food-environment and the individual-based or activity-based food environment [16,31,32,46,47,48,49]. Because food activities are not restricted by the delineation of geographic units, such as census tracts or community boundaries, individuals may be exposed to a series of food opportunities in their daily travel, and these opportunities could reach far beyond their residential neighborhood [46]. This issue, as a result of the SDMB, suggests that using place-based measures of the community food environment (e.g., the number of food stores in a census tract) may misrepresent the true food-environment exposure and the related associations with dietary health indicators [33,45]. In addition, relying on residential boundaries to define food environments is subject to the uncertain geographic-environment problem (UGCoP) [50], meaning that geographically delineated food-environment indicators may obfuscate the spatial scope and time frame of an individual’s exposure to the food environment [19,32]. To this end, the mobility data-driven approach can provide more nuanced information on the exposure to FAFH in people’s daily travel [50]. Leveraging this emerging dataset to design food-environment measures can provide insights into the environmental drivers and pathways through which community food environments affect health.

Our second finding shows that the mobility pattern in food procurement differs according to socioeconomic variables. The results are consistent with previous findings, which show that residents in socially vulnerable areas have fewer resources, such as reliable transportation, to travel for food [51]. Our finding further corroborates this conclusion by substantiating the interplay between the community food environment and social determinants of health. Specifically, findings related to how restaurant-visit patterns are influenced by neighborhood SVI can help researchers understand how socioeconomic inequities may shape health inequities, which has policy implications for designing an equitable community food environment. By considering residents’ mobility patterns and their determinants in the food environment, health researchers and policymakers can offer more precise and contextualized health promotion and intervention strategies. For example, combining large-scale mobility data with data on the nutritional quality of the food retailers, researchers can more accurately measure food procurement and diet quality of the residents. Through tracking residents’ mobility patterns, policymakers can identify activity-based food deserts or food swamps, which may be significantly different from the place-based ones. Policymakers could use these new methods in designating which areas are appropriate for federal, state, or local-development funding (for example, through the Healthy Food Financing Initiative [52]). The finding that restaurants located in socially vulnerable areas attracted more customers as well as more local customers indicated that restaurants in socially vulnerable areas could be significant local dietary-health drivers. In considering health-promoting policies related to commercial zoning laws or local food-ordinances, targeting these areas could reduce diet-related disparities. Improving the nutritional quality of these restaurants should be prioritized in planning initiatives and health interventions. For example, an ongoing initiative in Hartford is working with local restaurants to modify current recipes to reduce both sodium and saturated fat, and increase whole grains, fruits, and vegetables [53].

The study has limitations. First, the mobility data used in this study were aggregated by customers’ home census tracts. This limitation cannot be easily overcome, as SafeGraph data were collected anonymously, without the inclusion of individual characteristics. Thus, translating these findings from an aggregate level into individual implications might be ecologically fallible. Second, we focused on the Greater Hartford region as a case study, and thus our results might not be generalizable to other study areas. Third, while we controlled for many restaurant-level and census tract-level characteristics, the established correlations should be interpreted with caution. To better establish causal relationships, future studies should collect longitudinal data with a rich set of covariates and should also corroborate the findings with qualitative inquiries, such as food diaries and interviews. Finally, we intentionally only included data from 2018 to 2019, to avoid the impact of the COVID-19 pandemic. There may be significant changes in food-procurement activities since the pandemic (e.g., an increase in food deliveries [54], which is generally not captured in SafeGraph mobility data). Future studies could use more recent data from multiple sources to investigate the impact of the pandemic on food-procurement activities.

## 5. Conclusions

Using large-scale mobility data on restaurant patronization in the Greater Hartford region, this study demonstrates the existence of the SDMB in the community food environment, and identifies important associations with sociodemographic variables. These results demonstrate the necessity of incorporating human-mobility data into the study of the community food environment. They also hold much potential in offering valuable tools for health policymakers to design more contextualized initiatives and interventions. Future studies should continue this line of research by incorporating related mobility components into the discussion of food-environment exposure and dietary health.

## Figures and Tables

**Table 1:** Description of variables of interest.

	N (Count)	Min/Max	Mean (SD)	Median [IQR]
Median distance customers traveled	396	0.66/10.10	3.55 (1.99)	2.87 [2.25–4.41]
Percentage of customers originating from the same census tract as the restaurant location	396	0.00/50.00	8.41 (5.84)	7.14 [4.24–11.17]
Percentage of customers originated from within a 1-mile radius of the restaurant location	396	0.00/75.00	18.87 (11.79)	16.72 [10.21–26.70]
The SVI of the census tracts customers originated from (weighted by the number of visits)	396	0.32/0.87	0.61 (0.14)	0.59 [0.50–0.73]
The SVI of the census tract where the restaurant is located	396	0.02/1.00	0.59 (0.29)	0.51 [0.36–0.90]
Review counts	396	1.00/1260.00	86.00 (132.00)	37.00 [13.00–105.00]
Rating	396	1.00/5.00	3.50 (0.85)	3.50 [3.00–4.00]
Total population of the census tract where the restaurant is located	396	907.00/6581.00	3821.00 (1356.00)	3492.00 [2681.00–4683.00]
Total visit count in 2018–2019	396	16.00/14,686.00	2831.00 (2447.00)	2204.00 [1292.00–3593.00]
	N (count)	Percentage		
**Restaurant category**				
Limited-service restaurant (0)	327	82.58%		
Full-service restaurant (1)	69	17.42%		
**Average cost per person for a meal in a restaurant**				
Under USD 10 (1)	181	45.71%		
USD 11–30 (2)	208	52.53%		
USD 31–60 (3)	7	1.77%		
**Restaurant located in an urban tract**				
No (0)	3	0.76%		
Yes (1)	393	99.24%		
**Restaurant located in a food-desert census tract**				
No (0)	282	71.21%		
Yes (1)	114	28.79%		

**Table 2:** The results of multivariate-linear-regression models.

	Percentage of Customers from the Same Census Tract as the Restaurant They Visit (%)	Percentage of Customers within a 1-Mile Radius of the Restaurant Location	SVI of the Visitors’ Home Census Tract	Median Distance Traveled	Total Visit-Count of the Restaurant (Log-Transformed)
SVI of the census tract where the restaurant is located	0.33 [−1.79, 2.44]	16.61 *** [12.51, 20.72]	0.28 *** [0.25, 0.32]	−1.96 *** [−2.67, −1.25]	0.45 * [0.05, 0.84]
Total population of the census tract where the restaurant is located	1.37 × 10^−3^ *** [9.78 × 10^−4^, 1.76 × 10^−3^]	6.09 × 10^−4^ [−1.51 × 10^−4^, 1.37 × 10^−3^]	−1.07 × 10^−5^ ** [−1.73 × 10^−5^, −4.07 × 10^−6^]	−2.91 × 10^−4^ *** [−4.22 × 10^−4^, −1.59 × 10^−4^]	−2.42 × 10^−5^ [−9.73 × 10^−5^, 4.89 × 10^−5^]
Restaurant located in an urban tract	3.14 [−2.74, 9.03]	15.80 ** [4.39, 27.20]	0.09 [−0.01, 0.19]	−2.62 ** [−4.60, −0.65]	0.27 [−0.82, 1.37]
Restaurant located in a food-desert tract	2.48 *** [1.13, 3.83]	−0.23 [−2.85, 2.38]	0.04 ** [0.01, 0.06]	0.08 [−0.37, 0.53]	−0.09 [−0.34, 0.16]
Full-service restaurant	−1.27 [−2.69, 0.16]	−0.14 [−2.89, 2.62]	−0.02 [−0.05, 3.16 × 10^−3^]	0.19 [−0.29, 0.66]	−0.06 [−0.32, 0.21]
Review counts (log-transformed)	−0.92 *** [−1.35, −0.50]	−2.33 *** [−3.16, −1.50]	−0.02 *** [−0.03, −0.01]	0.33 *** [0.18, 0.47]	0.17 *** [0.09, 0.25]
Price	0.16 [−0.90, 1.22]	0.68 [−1.37, 2.74]	−0.01 [−0.03, 6.47 × 10^−3^]	0.39 * [0.04, 0.75]	−0.08 [−0.28, 0.12]
Rating	0.81 * [0.18, 1.44]	0.80 [−0.43, 2.03]	−1.48 × 10^−3^ [−0.01, 9.24 × 10^−3^]	0.07 [−0.14, 0.28]	−0.25 *** [−0.37, −0.14]
Constant	−0.43 [−6.55, 5.68]	−4.41 [−16.27, 7.45]	0.48 *** [0.38, 0.59]	6.35 *** [4.29, 8.40]	7.74 *** [6.60, 8.88]

95% CI in square brackets *** *p* < 0.001 ** *p* < 0.01 * *p* < 0.05.

## Data Availability

Data are available upon request.
